# An in-vitro three-dimensional surgical simulation technique to predict tibial tunnel length in transtibial posterior cruciate ligament reconstruction

**DOI:** 10.1186/s12938-024-01253-9

**Published:** 2024-06-17

**Authors:** Gengxin Jia, Xiaoyang Jia, Minfei Qiang, Tianhao Shi, Qinghui Han, Yanxi Chen

**Affiliations:** 1 Department of Orthopedic Surgery, Zhongshan Hospital, Fudan University, 180 Fenglin Rd, Shanghai, 200032 China; 2 Department of Orthopedic Trauma, East Hospital, Tongji University School of Medicine, 150 Jimo Rd, Shanghai, 200120 China

**Keywords:** Posterior cruciate ligament reconstruction, Tibial tunnel length, Popliteal neurovascular structure, Iatrogenic injury, 3D simulation

## Abstract

**Background:**

During the transtibial posterior cruciate ligament (PCL) reconstruction, drilling depth excessively longer than the tibial tunnel length (TTL) is an important reason to cause popliteal neurovascular bundle injury when preparing the tibial tunnel. This study aims to develop an in-vitro three-dimensional surgical simulation technique to determine the TTL in anteromedial (AM) and anterolateral (AL) approaches.

**Methods:**

A total of 63 knees’ 3-dimensional (3D) computed tomography models were included in this study. The SuperImage system was used to reconstruct the 3D knee model and locate the tibial PCL site. The established 3D knee model and the coordinates of the tibial PCL site were imported into Rhinoceros 3D modeling software to simulate AM and AL tibial tunnel approaches with different tibial tunnel angles (TTA). The TTL and the tibial tunnel height (TTH) were measured in this study.

**Results:**

In AM and AL tibial tunnel approaches, the TTL showed a strong correlation with the TTA (for AM: r = 0.758, p < 0.001; for AL: r = 0.727, p < 0.001). The best fit equation to calculate the TTL based on the TTA was *Y* = 1.04*X* + 14.96 for males in AM approach, *Y* = 0.93*X* + 17.76 for males in AL approach, *Y* = 0.92*X* + 14.4 for females in AM approach, and *Y* = 0.94*X* + 10.5 for females in AL approach.

**Conclusion:**

Marking the TTL on the guide pin or reamer could help to avoid the drill bit over-penetrated into the popliteal space to damage the neurovascular structure.

**Supplementary Information:**

The online version contains supplementary material available at 10.1186/s12938-024-01253-9.

## Introduction

Transtibial posterior cruciate ligament (PCL) reconstruction is a technically demanding procedure, and one of the most feared complications during the surgery is injury to the popliteal neurovascular bundle [1, 2]. Although the incidence of the neurovascular damage in transtibial PCL reconstruction is rare, the consequences can be devastating [3–5]. Clinically, drilling depth excessively longer than the tibial tunnel length (TTL) is an important reason to cause this intraoperative complication when preparing the tibial tunnel [5–7].

**Fig. 1:**
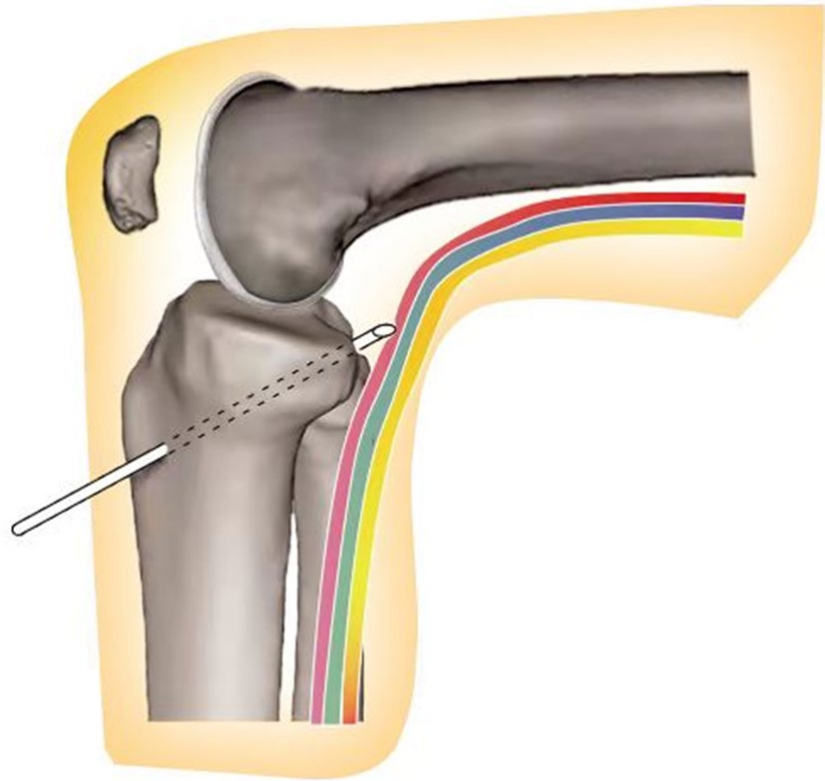
Schematic of the damage on the neurovascular bundle by the guide pin advanced inadvertently

On the anatomical level, the popliteal neurovascular bundle lies in proximity to the tibial PCL footprint. Hence, the popliteal neurovascular bundle is susceptible to potential risk if the guide pin or reamer advances excessively during transtibial PCL reconstruction (Fig. 1) [3, 8, 9]. To avoid the drill bit intersecting with neurovascular tissues in the popliteal fossa during tibial tunnel preparation, it is essential to align the drilling depth with the TTL. In clinical practice, directly visualizing the bit of guide pin or reamer at the PCL tibial footprint under the arthroscope from the anterior portals could be used to assure safety of the popliteal neurovascular bundle [10]. However, the surgeons may not know when the drill’s advancement speed should be further limited before the drill bit is visualized under the arthroscope [11]. Theoretically, to avoid the guide pin or reamer inadvertently over-penetrating into the popliteal space, surgeons should pre-limit the drill’s advanced speed before the bit penetrates the bone cortex. If the surgeon has no mental estimate of the drilling depth, the sharp drill might not stop advancing immediately as the drill bit exits the tibial posterior cortex [2].

Recently, suggestions to protect the popliteal neurovascular bundle during the transtibial PCL reconstruction have consistently focused on using the anterolateral tibial tunnel approach, or making the drill bits safer [3, 5, 11–14]. Actually, knowing the TTL value before drilling the tibial tunnel could remind surgeons when to limit the drilling speed to prevent the drill bit from excessively passing through the exit [3, 15]. However, few studies have reported the TTL and provided an optimal tibial tunnel drilling depth for the transtibial PCL reconstruction. In this study, the purposes were to: (1) develop an in-vitro three-dimensional surgical simulation technique to determine the TTL of the anteromedial (AM) and anterolateral (AL) approaches; (2) explore whether the tibial tunnel length is associated with anthropomorphic factors (sex, age, height, and BMI) of the patient; and (3) evaluate the correlation between the change in TTL and the variables of tibial tunnel angle (TTA) and tibial tunnel height (TTH).

## Results

According to the inclusion and exclusion criteria, 63 knees (31 males and 32 females) were included in this study. The average age of the patients was 34.1 ± 7.9 (ranged from 18 to 49) years, the average height of the patients was 167.4 ± 13 cm (ranged from 144 to 192 cm), and the average body mass index of the patients was 23.6 ± 3.7 kg/m^2^ (ranged from 15.9 to 32.9 kg/m^2^). In terms of TTL, the intra-observer ICC was 0.924 and the inter-observer ICC was 0.912. Therefore, consistency within and between observers were excellent in this study.

### Outcomes in AM and AL approaches

With a same TTA (from 40° to 60°), there was no differences in the mean TTH between the AM and AL approaches. However, in the AM approaches, the mean TTL was 2 to 3 mm longer compared to the AL approaches (Table 1). It should be noted that the mean value of TTL ranged from 54.9 mm to 74.6 mm for AM approaches, ranged from 52.7 mm to 71.5 mm for AL approaches. As the TTA increased, both TTH and TTL showed a significant increase (p < 0.001).

**Table 1:** Measurement’s outcomes of TTH and TTL in AM and AL groups

Parameter	TTH (mm)		p value	TTL (mm)		p value
	AM	AL		AM	AL	
40°	42.7 ± 4.1 (30.3 to 51.5)	41.8 ± 4.1 (30 to 51.1)	0.19	54.9 ± 4 (42.8 to 67.2)	52.7 ± 5.4 (37.8 to 67)	0.03
45°	48.7 ± 4.5 (58.8 to 35.4)	47.6 ± 4.5 (58.3 to 35.1)	0.18	58.2 ± 5.3 (45.8 to 71.4)	55.6 ± 5.6 (39 to 69.4)	0.01
50°	55.2 ± 5 (40.5 to 67.4)	53 ± 5.1 (40.1 to 66.9)	0.18	62.2 ± 5.4 (48.9 to 76.5)	59.4 ± 5.9 (43.2 to 74.9)	0.01
55°	62.7 ± 5.8 (46.2 to 77. 5)	61.5 ± 5.8 (45.8 to 76)	0.23	67.7 ± 6.3 (52.5 to 83.9)	64.7 ± 6.4 (47.5 to 82.3)	0.01
60°	71.6 ± 6.7 (53.5 to 88.7)	70.3 ± 6.8 (53.1 to 88.1)	0.28	74.6 ± 7.1 (58.1 to 92.8)	71.5 ± 7.2 (53.8 to 90.9)	0.02

TTH and TTL were expressed as mean ± standard deviation (range)

*AM* anteromedial approach; *AL* anterolateral approach; *TTL* length of the PCL tibial tunnel; *TTH* perpendicular distance of the tunnel entry point to the tibial plateau; 40° to 60°, 40° to 60° tibial tunnel angle

p value, AM group compared with the AL group

### Anthropomorphic factors

As the analysis outcomes showed in Table 2, the TTL in males was significantly longer than that in females in the AM and AL approaches (p < 0.001). The correlation analysis presented in Table 3 indicates that, within both the AM and AL approaches, there exists a moderate correlation between patients’ height and TTL (r ranging from 0.63 to 0.72). Additionally, the age of patients exhibits a weak correlation with TTL (r ranging from − 0.31 to − 0.25), while no significant correlation was observed between patients' BMI and TTL.

**Table 2:** TTL parameters in gender groups

Parameter	AM		p value	AL		p value
TTL (mm)	Male (n = 31)	Female (n = 32)		Male (n = 31)	Female (n = 32)	
40°	57.5 ± 4.6	52.3 ± 4.1	< 0.001	56.4 ± 4.5	49.2 ± 3.8	< 0.001
45°	61.1 ± 4.8	55.4 ± 4.3	< 0.001	59 ± 4.5	52.3 ± 4.6	< 0.001
50°	65.2 ± 4.6	59.2 ± 4.6	< 0.001	62.7 ± 4.9	56.1 ± 4	< 0.001
55°	71.2 ± 5.5	64.3 ± 5.2	< 0.001	68.2 ± 5.5	61.3 ± 5.4	< 0.001
60°	78.4 ± 6.5	70.9 ± 5.7	< 0.001	75.1 ± 6.9	68.1 ± 5.9	< 0.001

TTL was expressed as mean ± standard deviation

*AM* anteromedial approach; *AL* anterolateral approach; *TTL* length of the PCL tibial tunnel

40° to 60°, 40° to 60° tibial tunnel angle

p value, male group compared with the female group

**Table 3:** Correlation analysis between TTL and patient characteristics (height, age and BMI)

Parameter	Height versus length		Age versus length		BMI versus length	
	r value	p value	r value	p value	r value	p value
40° (AM)	0.63	< 0.001	− 0.25	0.05	0.15	0.23
45° (AM)	0.65	< 0.001	− 0.28	0.03	0.15	0.23
50° (AM)	0.63	< 0.001	− 0.27	0.04	0.15	0.25
55° (AM)	0.67	< 0.001	− 0.27	0.03	0.16	0.2
60° (AM)	0.66	< 0.001	− 0.27	0.03	0.17	0.18
40° (AL)	0.72	< 0.001	− 0.31	0.01	0.07	0.61
45° (AL)	0.69	< 0.001	− 0.31	0.01	0.09	0.5
50° (AL)	0.67^ s^	< 0.001	− 0.28^ s^	0.03	0.11^ s^	0.4
55° (AL)	0.66^ s^	< 0.001	− 0.26^ s^	0.04	0.14^ s^	0.27
60° (AL)	0.64	< 0.001	−0.25	0.05	0.17	0.19

*AM* anteromedial approach; *AL* anterolateral approach; *TTL* length of the PCL tibial tunnel; 40° to 60°, 40° to 60° tibial tunnel angle; *BMI* body mass index

^S^ Spearman’s correlation coefficient; Pearson’s correlation coefficient has no superscript

### Correlation between TTL and the variables of TTA and TTH

The TTL in both the AM and AL approaches showed a strong correlation with the TTA (for AM: r = 0.758, p < 0.001; for AL: r = 0.727, p < 0.001), and the TTH (for AM: r = 0.954, p < 0.001; for AL: r = 0.941, p < 0.001).

### Linear regression analysis

**Fig. 2:**
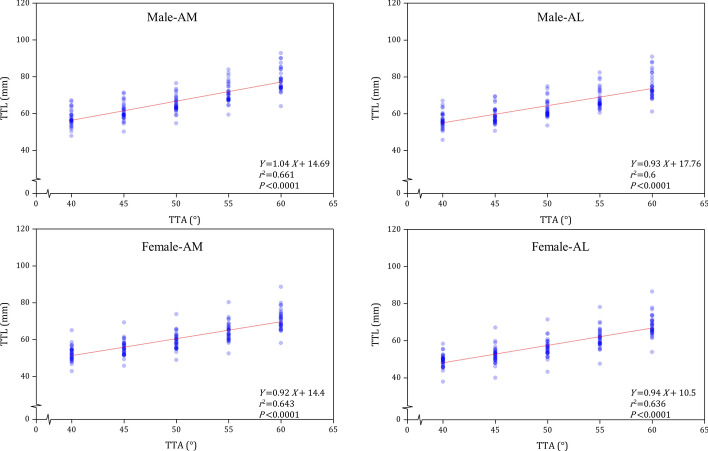
The linear relationship and the best-fit equations between the TTA and TTL in AM and AL tibial tunnel approaches for males and females

**Fig. 3:**
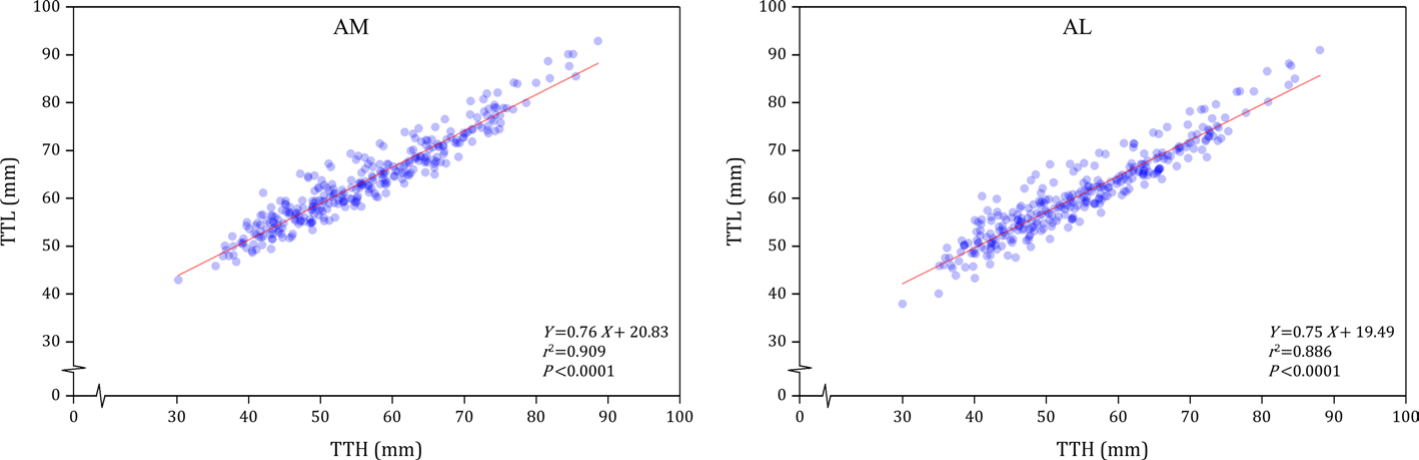
The linear relationship and the best-fit equations between the TTH and TTL

In AM and AL approaches, the TTA and TTL had a significant proportional relationship. The best-fit equation for calculating TTL with TTA was *Y* = 1.04*X* + 14.69 for males in AM approach, *Y* = 0.93*X* + 17.76 for males in AL approach, *Y* = 0.92*X* + 14.4 for females in AM approach, and *Y* = 0.94*X* + 10.5 for females in AL approach (Fig. 2). The calculated probability of no damage to the neurovascular bundle was 99.1% for males in AM approach, 98.7% for males in AL approach, 98.8% for females in AM approach, and 98.8%, for females in AL approach. As Fig. 3 showed, there is a significant proportional relationship between the TTL and TTH, the best-fit equation for calculating TTL with TTH was *Y* = 0.76*X* + 20.83 in AM tibial tunnel approach, and *Y* = 0.75*X* + 19.49 in AL tibial tunnel approach.

## Discussion

This study has developed an in-vitro three-dimensional surgical simulation technique to analyze the TTL in transtibial PCL reconstruction. The results have shown that TTL significantly varies as the tibial tunnel approach changed. Furthermore, TTL exhibits a strong linear relationship with the TTA and TTH. The best-fit equations could be used to calculate the TTL by utilizing the TTA and TTH during the PCL reconstruction, which enabling the surgeons to evaluate the drilling depth while preparing the tibial tunnel.

### The importance of TTL in transtibial PCL reconstruction

TTL determined the drilling depth during preparing the tibial tunnel in PCL reconstruction. In this study, in both the AM and AL approaches, the mean value of TTL varies by approximately about 20 mm when TTA changes from 40° to 60°. This difference is quite large for the transtibial PCL reconstruction, as the total TTL is only a few tens of millimeters [6]. In accordance with previous studies, recommended TTAs have been documented within the range of 42° to 70° [12, 16–18], signifying the potential for greater variability in the corresponding TTL within clinical practice. Additionally, this study demonstrates that under the same TTA, the TTL in males is significantly longer than in females, which may be attributed to the typically thicker proximal tibia in males than in females. Consequently, to avoid the guide pin or reamer being advanced excessively and inadvertently, it is important for surgeons to know these significant differences and refer to the different TTLs according to different tibial tunnel approaches and individuals. Without a referenced TTL value as a warning sign for surgeons, the drilling procedure might not be stopped promptly when the drill bit is exits the bone cortex.

### The method to predict the TTL

Clinically, the TTA is used to calibrate the PCL drill system [19, 20]. In order to provide a referenced tibial tunnel drilling depth for the transtibial PCL reconstruction, we have explored the relationship between the TTA and TTL. Fortunately, the TTA has demonstrated a significant proportional relationship with TTL. It is suggested that the TTA could be used to predict the TTL during the PCL reconstruction. The best-fit equation to calculate the TTL based on the TTA was *Y* = 1.04*X* + 14.96 for males in AM approach, *Y* = 0.93*X* + 17.76 for males in AL approach, *Y* = 0.92*X* + 14.4 for females in AM approach, and *Y* = 0.94*X* + 10.5 for females in AL approach. When preparing the tibial tunnel, the predicted TTL could be marked on the guide pin and reamer to remind surgeons when the drilling should be slower and more careful to avoid the drill bit inadvertently over-penetrate into the popliteal space.

Since the correlation between TTL and TTA is not 100%, the predicted TTL may be longer or shorter than the actual value in some patients. If the predicted TTL is longer than the actual value, the drill may penetrate the posterior tibial cortex. Therefore, this study further evaluated the safety rate of using the equations to calculate TTL. The results show that the calculated probability of no damage to the neurovascular bundle was 99.1% for males in AM approach, 98.7% for males in AL approach, 98.8% for females in AM approach, and 98.8%, for females in AL approach. Therefore, the slowing down or cessation of drilling prior to reaching the predicted TTL, coupled with arthroscopic observation, enables surgeons to effectively mitigate the risk of injuries.

Clinically, arthroscopic visualization of the tibial PCL footprint has been commonly used to ensure a safe tibial tunnel placement [10, 14]. However, the tibial footprint may not always be visualized under the arthroscope. To verify that the pin has not intersected with the popliteal neurovascular bundle, intraoperative fluoroscopy is commonly used to confirm the correct location of the guide pin or reamer [3, 13]. However, frequent use of intraoperative fluoroscopy exposes doctors and patients to repeated radiation, and the operative time might also be prolonged. Based on the results of the present study, the guide pin or reamer could initially drill to a depth nearing the predicted TTL, then intraoperative fluoroscopy can be used to confirm the location of the drill bit. In this way, in addition to ensuring safety, the number of intraoperative fluoroscopies could be reduced and the operation time could be shortened.

### The relationship between TTH and TTL

In this study, TTH is found to be significantly correlated with TTL. Therefore, TTH could also be used to predict TTL during transtibial PCL reconstruction. According to the outcomes of regression analysis, the relationship between the TTH and TTL is closer than that between the TTA and TTL. However, due to the influence of soft tissue, TTH could be only roughly measured on the body surface during preparing the tibial tunnel [6]. For clinical practice, the use of TTA to predict the TTL is a simpler and more practical approach. In the future, custom tibial guides for each patient might be established preoperatively, and using TTH to calculate TTL based on computer software could provide a more accurate reference.

### The TTL in AM and AL approaches

Several studies have revealed that the AL tibial tunnel approach could be used to avoid the guide pin intersecting with the popliteal neurovascular bundle [10]. Nevertheless, according to the study of Franciozi et al. [5], the distance between the pin exit and popliteal artery at tibial posterior cortex projected at tibial level in AL approach is found to be closer than the AM approach. Although the guide pin is proved that did not threaten the popliteal artery with the usage of AL tibial tunnel approach, the reamer might still could (the reamers are wider than pins) [5]. In current study, the TTL in the AL tibial tunnel approach is found to be 2 to 3 mm shorter than the AM approach. The result suggests that the safe distance between the tibial tunnel entry point and the popliteal neurovascular bundle is shorter in the AL approach. Consequently, while using the reamer, it’s essential for surgeons to note this difference and master the drilling depth more carefully in AL approach.

Other methods to make this surgery safer were: using the spade-tipped guidewire, the oscillating drill, and the tapered drill bit, but these methods only alleviated the severity of the neurovascular injury, and could not avoid the damage [2, 11, 21]. Consequently, using the estimation equation to determine the TTL in our study is an important and feasible method to obtain the TTL, which could be used to protect the neurovascular structure in popliteal fossa during drilling the tibial tunnel fundamentally.

## Limitations

(1) This was a theory study by using the 3D knee model, we did not consider the impact of the soft tissues. Therefore, the outcomes need to be validated by future clinical or cadaveric studies. Previous studies have demonstrated that 3D knee joint models can be used for precise quantitative analysis and exhibit excellent measurement accuracy and reliability [22]. Therefore, the results of this study can facilitate surgeons in better preoperative planning and intraoperative estimation of TTL. (2) The tibial PCL attachment site was located by using the sagittal CT image, which might raise a concern about precision. We have manually adjusted the grayscale value of the CT image, and referred to the digital definition of the center of the PCL anterolateral and postmedial bundles’ tibial insertion by Osti et al.[23]. Therefore, the definition of PCL attachment center was accurate. (3) The patients who had PCL injuries were excluded by us. This might be a potential limitation, because a decreased posterior tibial slope might be associated with patients who were prone to PCL tears [24], while the TTL is related to the position of the tibial slope. We must use a normal knee for the 3D modeling, because a rupture on the PCL might result in an unclearly tibial PCL attachment in the CT image. Besides, the effect on the TTL caused by the patients who were prone to PCL tears was slight. (4) The TTL showed a strong correlation with the TTA but was not 100% perfect. However, this study did not ask for the surgeons must follow the equation strictly to drill the tibial tunnel. It told the surgeons to be slower and more careful when the drilling depth was close to the calculated value during drilling the tibial tunnel. Moreover, prediction of TTL combined with arthroscopic observation can avoid iatrogenic injuries on the neurovascular bundle maximumly.

## Conclusions

TTL significantly varied with different surgical approaches. With the same TTA, TTL in AL tibial tunnel approach was shorter than in AM approach. The best-fit equation for calculating TTL with TTA was *Y* = 1.04*X* + 14.96 for males in AM approach, *Y* = 0.93*X* + 17.76 for males in AL approach, *Y* = 0.92*X* + 14.4 for females in AM approach, and *Y* = 0.94*X* + 10.5 for females in AL approach. Marking the TTL on the guide pin or reamer could help avoid the drill bit from over-penetrating into the popliteal space and damaging the neurovascular structure.

## Methods

### Sample selection

Local institution’s ethics committee granted approval for this study. The computed tomography (CT) images (June 2019 to January 2021) of 63 knee joints were selected from the CT database in our hospital for review. Inclusion criteria encompassed (1) Kellgren-Lawrence grade of knee osteoarthritis less than 1 [25]; (2) patients age ranging from 18 to 60 years; (3) clear identification of tibial PCL attachment in CT images. Patients with knee deformities, a history of knee surgery, fractures, or soft tissue injuries around the knee were excluded.

### Establishment of 3D knee model

The routine clinical knee CT of all included patients were conducted using a 64-multidetector-row CT (SOMATOM Sensation, Siemens AG, Wittelsbacherplatz 2, Muenchen, Germany). Scanning parameters comprised a gantry rotation speed of 1.00 s/rotation, a collimation width of 0.625 mm × 12 detectors, a CT pitch factor of 0.90, and a field of view of 25–30 cm. The CT dose index (CTDI) volume was 20.9 mGy. SuperImage system (orthopedic edition 1.1; Cybermed), a computer-assisted orthopedic clinical research platform, was used to process the Digital Imaging and Communications in Medicine (DICOM) format of the knee’s CT image for establishing the 3D model of the knee joint. The Rhinoceros software (Rhino 7, Robert McNeel and Associates for Windows, Washington DC, USA) was utilized to process the 3D knee model and simulate the surgical procedure of the tibial tunnel preparation in PCL reconstruction.

### Method to place the tibial tunnels of transtibial PCL reconstruction

The methods to determine the center of the PCL attachment on the tibia was referred from previous studies [6, 26]; The detailed steps were as follows: First, select a CT image that displays the tibial PCL attachment clearest and widest in the sagittal plane. Second, manually adjust the grayscale value of the CT image to display the PCL attachment more clearly. Third, mark a point on the observed PCL tibial attachment center on the sagittal CT image, which will also appear in the 3D view at the same time. Fourth, read the 3D coordinate of the PCL attachment center from the SuperImage system, and input it into the Rhinoceros software. In this way, the Rhinoceros software will automatically generate the location of the tibial PCL attachment center.

**Fig. 4:**
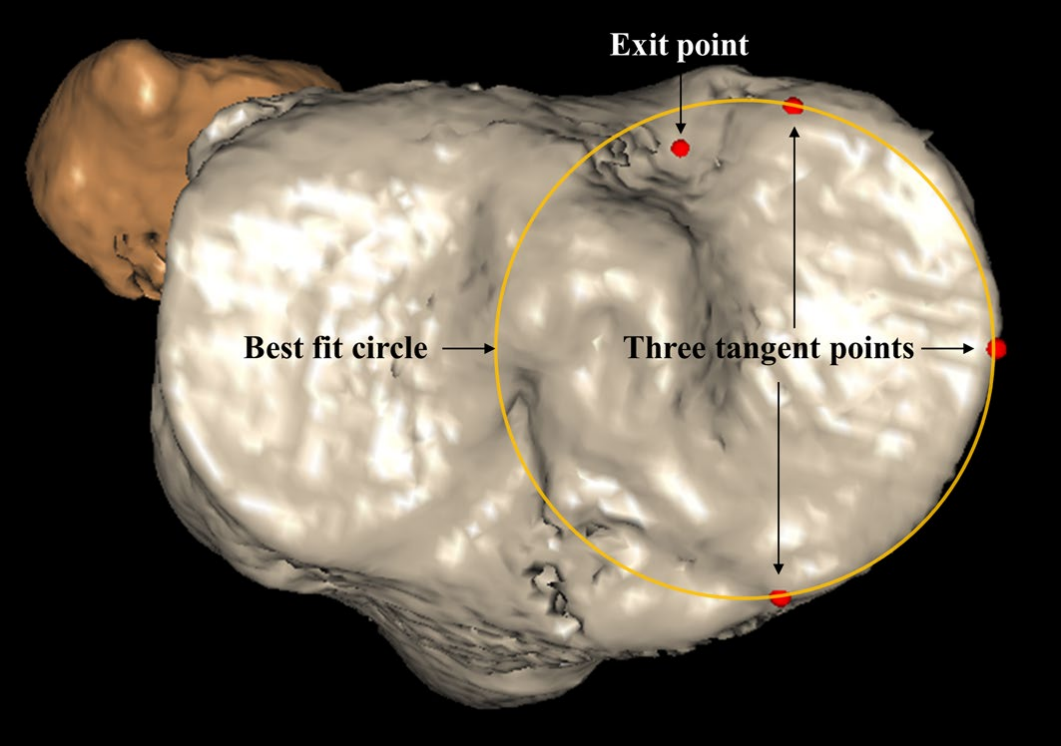
The method of best fit circle to determine the tibial plateau. Three tangent points: the most anterior point of the medial tibial plateau; the most medial point of the tibial plateau; the most posterior point of the medial tibial plateau

The medial tibial plateau was established as the reference plane to determine the entry point of the tibial tunnel [27, 28]; A best-fit circle that tangent to the cortical edge of the medial tibial plateau was used to produce three tangent points (the most anterior point of the medial tibial plateau; the most medial point of the tibial plateau; the most posterior point of the medial tibial plateau) to determine the tibial plateau plane (Fig. 4) [25].

**Fig. 5:**
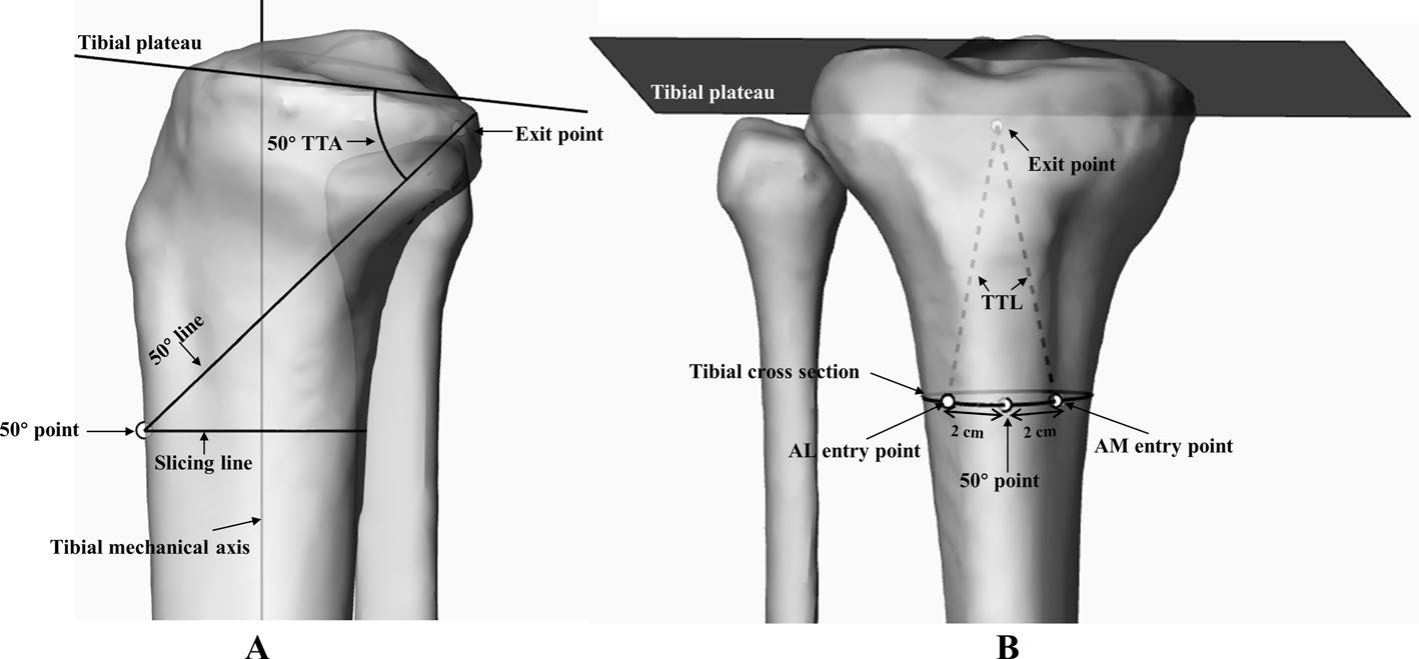
The method to simulate the transtibial PCL reconstruction. **A** The method to obtain the 50° oblique section and the 50° point tibial cross section. **B** The method to locate the tibial tunnel entry point of AM and AL approaches

**Fig. 6:**
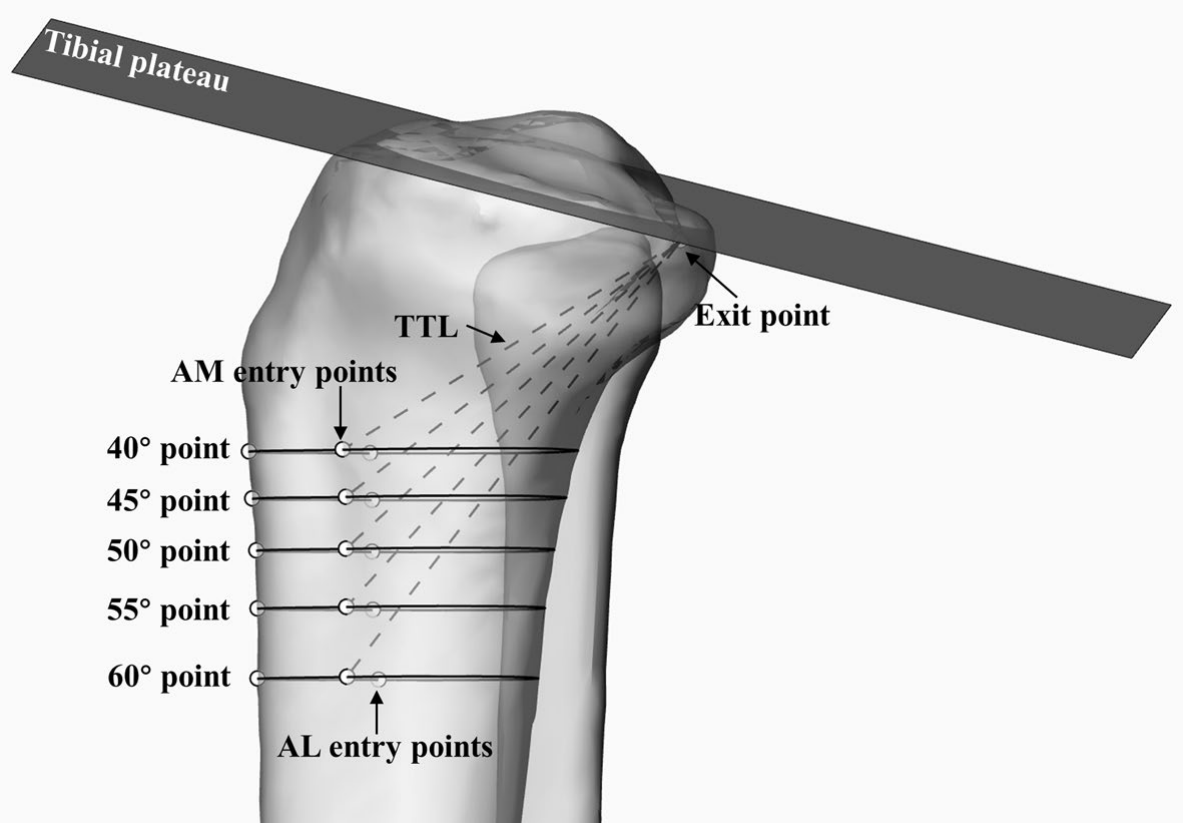
Different tibial tunnel entry points

The tibial tunnels in both the AL and AM approaches were simulated and analyzed in this study. In the lateral perspective, a line inclined at a 50° angle (50° TTA) in relation to the tibial plateau was drawn intersected the exit point of the tibial tunnel. This line was employed to section the tibia in the lateral view, subsequently revealing a 50° oblique section in the 3D perspective view. In the 3D perspective view, a point (50° point) was manually marked at the most anterior position of the tibial crest on the oblique tibial section (Fig. 5A). The AL and AM tibial tunnel entrance points were respectively placed at 2 cm posterolateral and posteromedial from the 50° point on the tibial cross section (Fig. 5B) [1, 5]. The above method was used continually to locate the AL and AM tibial tunnel entrance points by creating the 40°, 45°, 55° and 60° TTA, respectively (Fig. 6) (See additional file 1 for more detailed measurement procedures).

### Outcome measurements

**Fig. 7:**
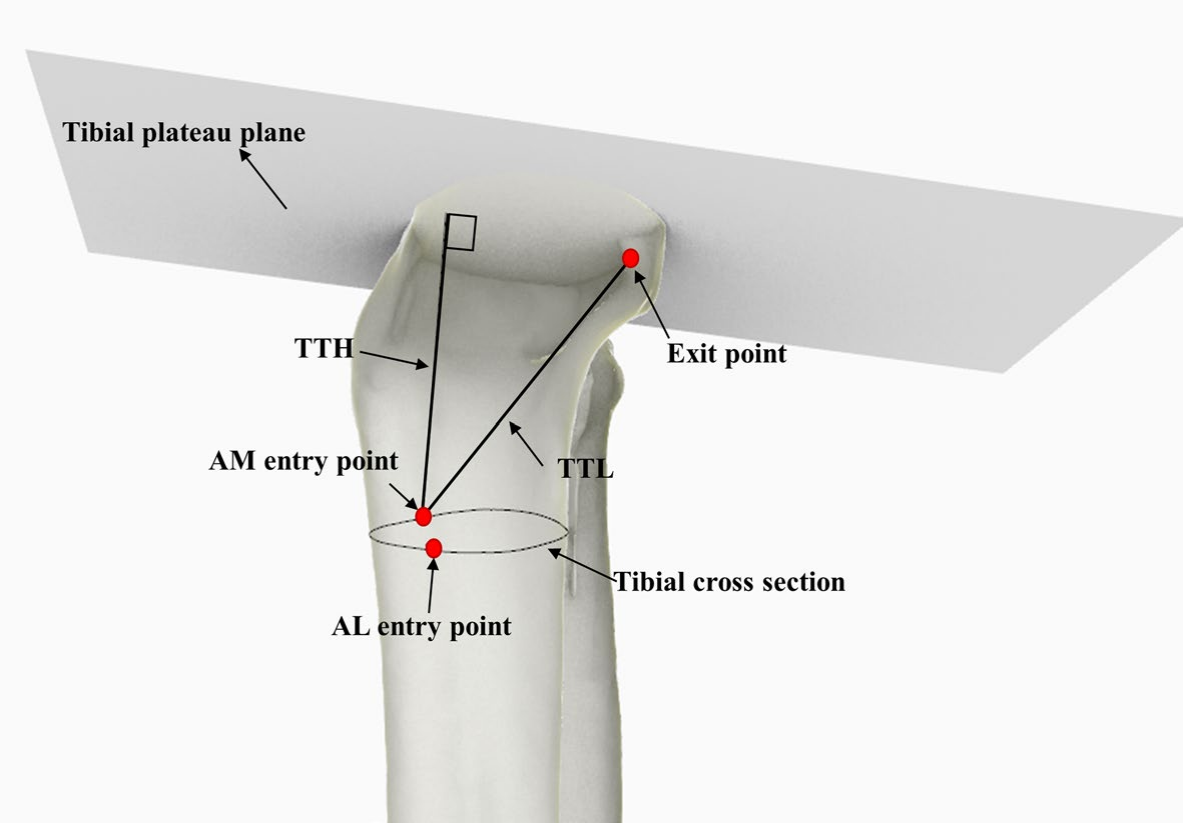
The measuring model to measure the TTH and TTL. The TTL was defined as the distance between the entry point and exit point of the tibial tunnel. The TTH was defined as the perpendicular distance from the tibial tunnel entry point to the tibial plateau plane

TTL and the TTH were measured in the study. TTL was characterized as the distance between the entry and exit points of the tibial tunnel, while TTH was characterized as the perpendicular distance from the tunnel entry point to tibial plateau plane (Fig. 7).

Two orthopedic surgeons conducted the measurements. Both surgeons have undergone standardized training to simulate the tibial tunnel preparation for PCL reconstruction. The full procedures from the initial 3D knee model establishment to the outcome measurement were performed two times by one surgeon at least 4 weeks apart to ensure intra-observer reproducibility. Another surgeon repeated the whole procedure to evaluate the inter-observer reproducibility. The intra- and inter-observer reproducibility were evaluated by intraclass correlation coefficients (ICCs). An ICC less than 0.50 indicated poor agreement, 0.50 to 0.75 was deemed moderate agreement, 0.75 to 0.90 was deemed good agreement, and greater than 0.90 was deemed excellent agreement [29].

### Statistical analysis

F test (ANOVA: Repeated measures; A priori) function of the G*Power software (version 3.1.9, Heinrich Heine University, Düsseldorf, Germany) was used to calculate the minimum sample size based on the pre-experiment data of different TTL in 40°, 45°, 50°, 55° and 60° TTA groups (AM tibial tunnels). By calculation, at least 25 samples were required for this study (effect size f = 0.75; 1-β err prob = 0.9; α err prob = 0.05). The data were analyzed using SPSS software (version 26.0, Inc., Chicago, IL, USA), and the findings were presented as mean ± standard deviation. Statistical comparisons between males and females, as well as between the AM and AL groups, were conducted using an independent t-test, and p < 0.05 was considered statistically significant.

Pearson’s correlation analysis, suitable for two normally distributed variables, or Spearman’s correlation analysis, applicable to non-normally distributed variables, was used to assess the relationships between TTL and patients’ anthropomorphic characteristics, including age, height, and BMI. The Kolmogorov–Smirnov test was used to evaluate whether the data obeyed the normal distribution. A correlation coefficient (r) in the range of 0.1 to 0.3 was considered a weak correlation, 0.3 to 0.7 indicated a moderate correlation, and 0.7 to 1.0 indicated a strong correlation [25]. Linear regression analysis to determine the relationship between TTL and TTA, TTL and TTH, as well as the identification of the best-fit equations, was conducted using GraphPad Prism software (version 9; GraphPad Software, Inc.). Coefficients of determination (r^2^) were utilized to identify the goodness of fit and predictive ability [30, 31]. As reported by Franciozi et al. [5], the distance between the popliteal artery and pin exit at the tibial posterior cortex ranged from 8 to 12 mm. To test the security rate of the equations calculated in this study, we have considered 8 mm as the safe distance between the popliteal artery and the pin exit at the tibial posterior cortex. Safety rate is defined as the probability of the drill bit not intersected with the neurovascular bundle within the popliteal fossa when using the equations.

### Supplementary Information

Additional file 1: Detailed steps to measure the TTL.

## Data Availability

All the data used and/or analyzed during the current study are available from the corresponding author upon reasonable request.

## Abbreviations

AL: Anterolateral
AM: Anteromedial
BMI: Body mass index
CT: Computed tomography
DICOM: Digital imaging and communications in medicine
3D: Three-dimension
ICC: Intraclass correlation coefficient
PCL: Posterior cruciate ligament
TTL: Tibial tunnel length
TTA: Tibial tunnel angle
TTH: Tibial tunnel height
