# Supplementary material for: An in-vitro three-dimensional surgical simulation technique to predict tibial tunnel length in transtibial posterior cruciate ligament reconstruction
Source: Biomed Eng Online. 2024 Jun 17;23:54. doi: 10.1186/s12938-024-01253-9 (PMC11181606; doi:10.1186/s12938-024-01253-9)
Supplement: Supplementary file 1 — Additional file 1: Detailed steps to measure the TTL. [file 12938_2024_1253_MOESM1_ESM.pdf]

Determination of the center of the PCL attachment and the medial tibial plateau by using SuperImage system

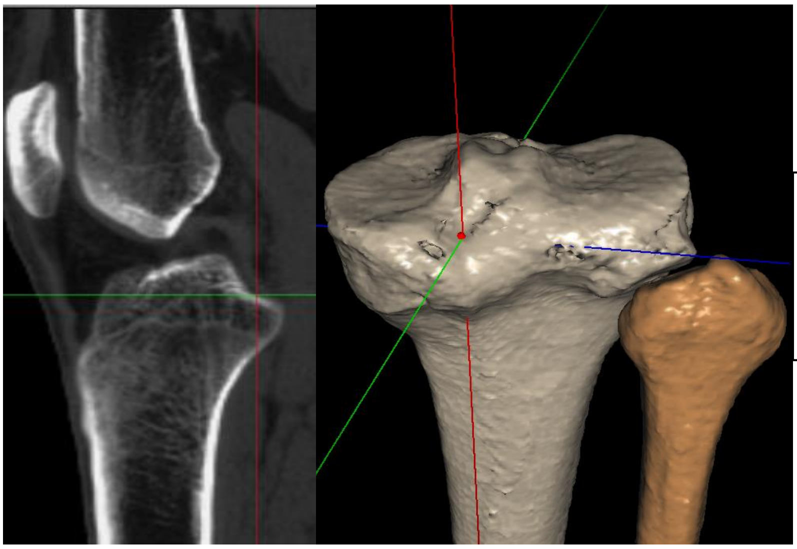

1. This study located the center point of the PCL attachment on the 3D knee model by using CT sagittal image on the SuperImage system.

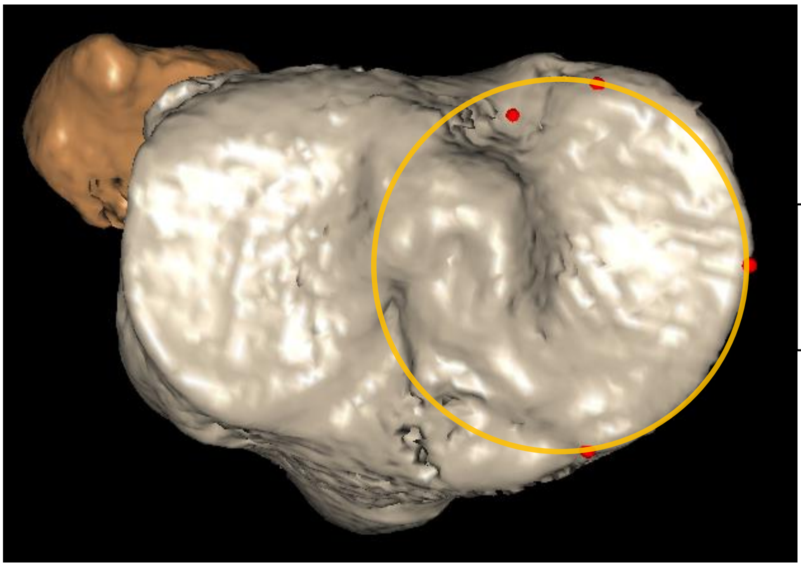

2. Using the method of the best fit circle to create medial tibial plateau on the SuperImage system.

Measurement process on Rhinocreos software

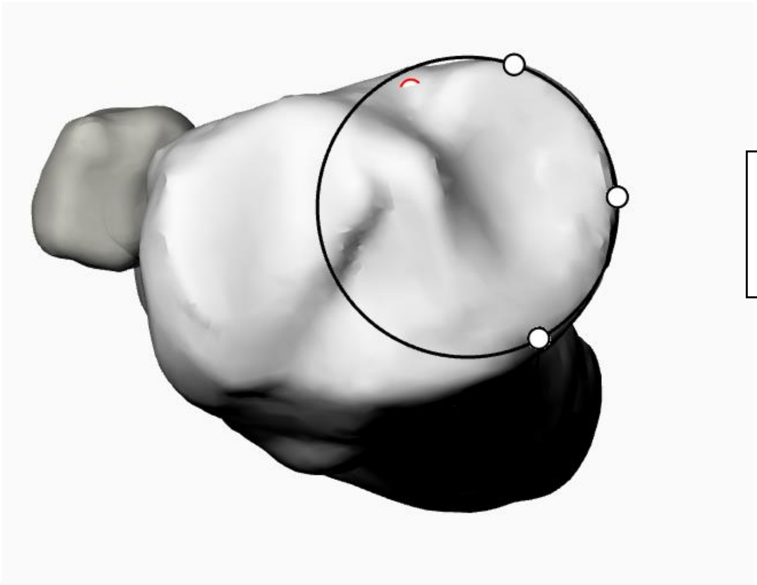

1. Using the method of the best fit circle to create medial tibial plateau. (red point: exit point).

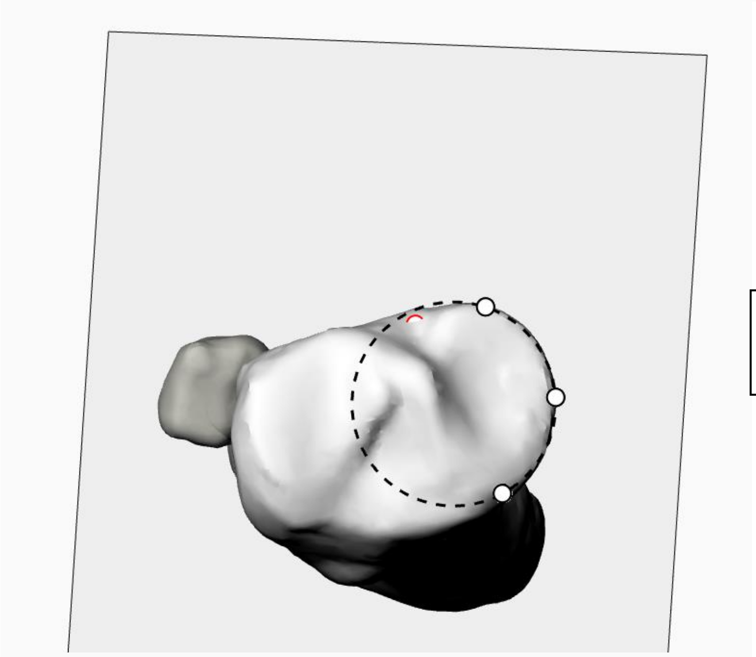

2. Formation of reference plane of tibial plateau.

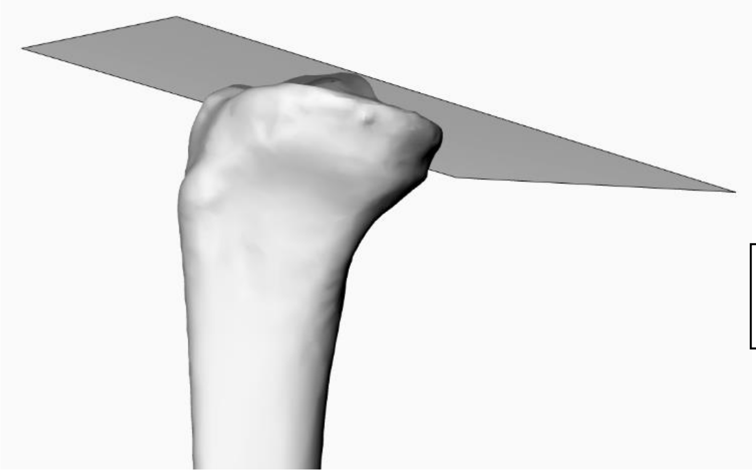

3. Lateral view of the generated tibial plateau plane.

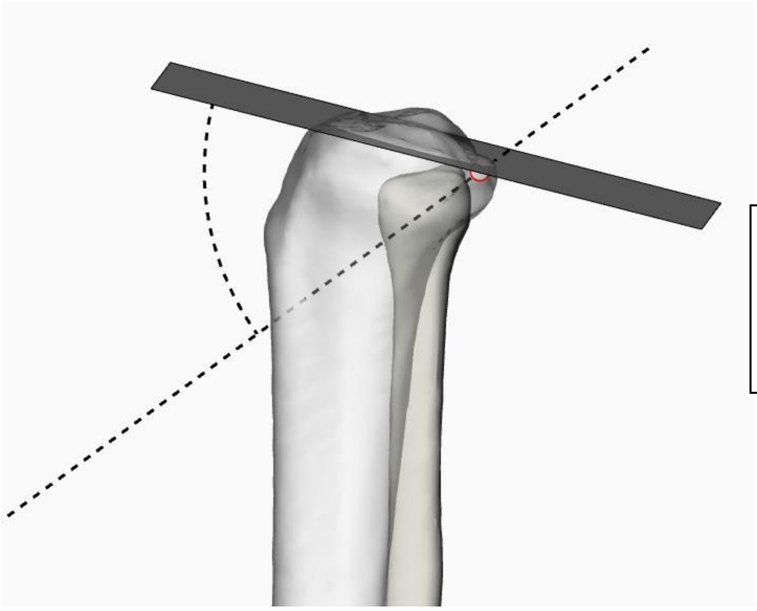

4. Crossing the tibial tunnel exit point, a line at an angle of 50° (50° TTA) relative to the tibial plateau plane was drawn in the lateral view.

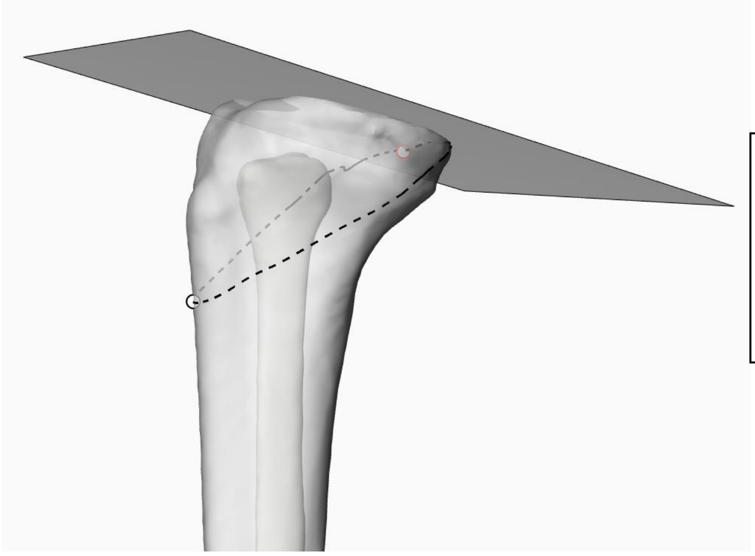

5. Using the line cut the tibia in the lateral view, then an oblique tibial section (50° oblique section) could be observed in the 3D perspective view (dotted line).

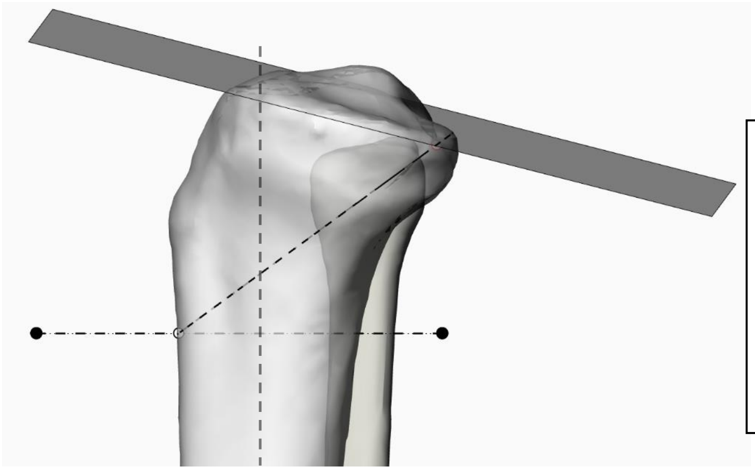

6. A point (50° point: black hollow point) was manually marked at the most anterior position of the tibial crest. Through this point, a line perpendicular to the mechanical axis of the tibia was used to cut the tibia.

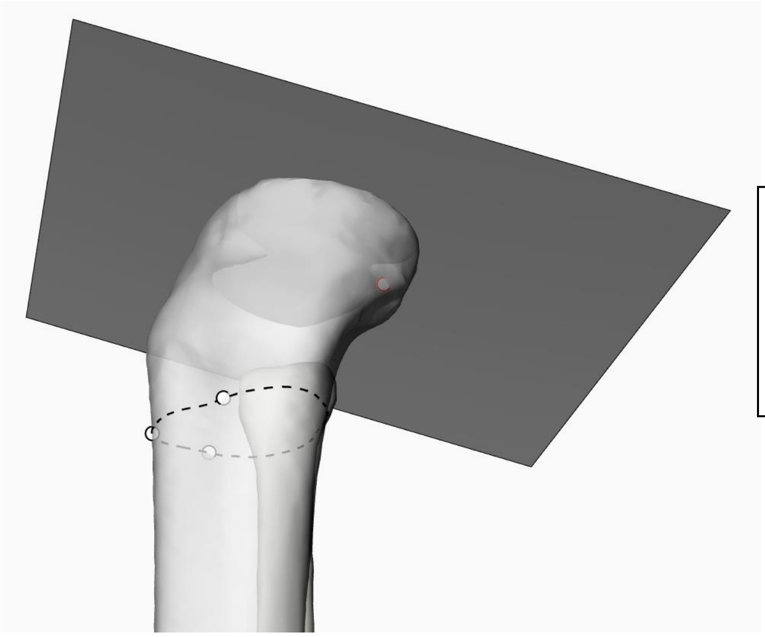

7. The AL and AM tibial tunnel entrance points were respectively placed at 2cm posterolateral and posteromedial from the 50° point on the tibial cross section.

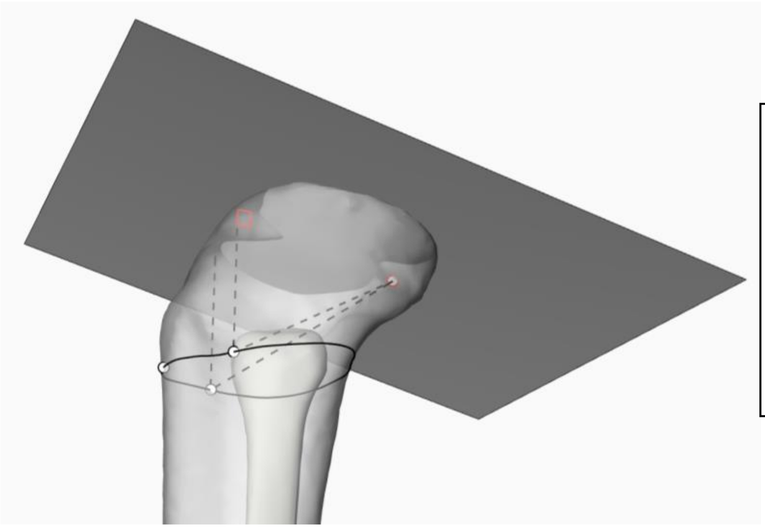

8. The TTL was defined as the distance between the entry point and exit point of the tibial tunnel; the TTH was defined as the perpendicular distance from the tibial tunnel entry point to the tibial plateau plane.

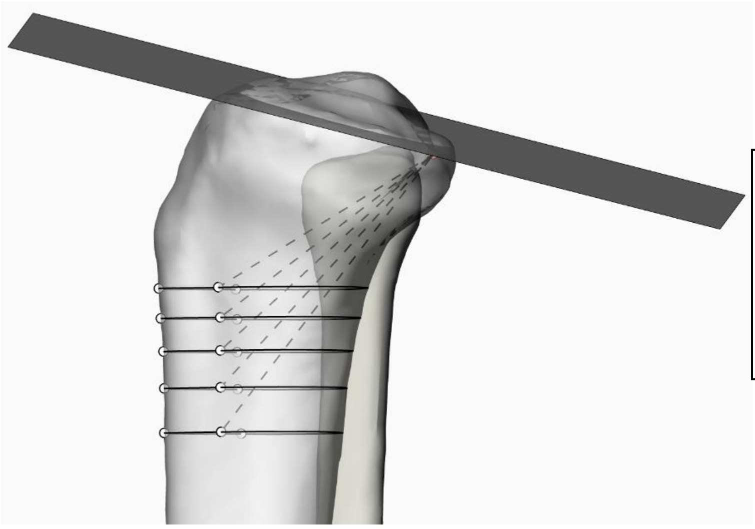

9. The above method was used continually to locate the AL and AM tibial tunnel entrance points by creating the 40°, 45°, 55° and 60° TTA, respectively.

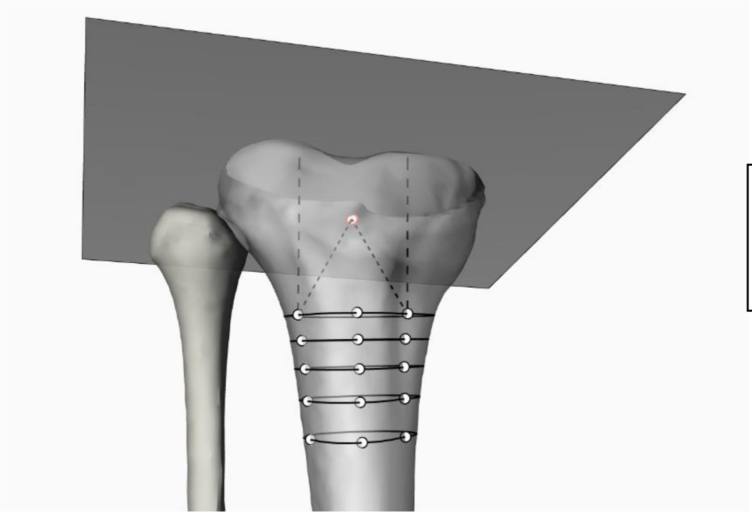

10. Perspective view to show the TTL and TTH of different entry point.

Note: All figures are directly exported from Rhino software.
